# 12 years of specialized pediatric palliative inpatient care: description of patients' characteristics

**DOI:** 10.3389/fped.2026.1915934

**Published:** 2026-08-28

**Authors:** Aleksandra Kosolapova, Dejan Vlajnic, Larissa Kubek, Christina Becker-Emden, Dörte Garske, Mandira Reuther, Deborah Wiesmann, Julia Wager, Boris Zernikow, Pia Schmidt

**Affiliations:** 1 Department of Children's Pain Therapy and Pediatric Palliative Care, Faculty of Health, School of Medicine, Witten/Herdecke University, Witten, Germany; 2 Pediatric Palliative Care Centre, Children's and Adolescents' Hospital Datteln, Datteln, Germany; 3 Department of Pediatric Palliative Care, Klinikum Leverkusen, Leverkusen, Germany; 4 PedScience Research Institute, Datteln, Germany; 5 German Pediatric Pain Centre, Children's and Adolescents' Hospital Datteln, Datteln, Germany

**Keywords:** inpatient, life-limiting conditions, multidisciplinary care, palliative care, pediatric

## Abstract

**Background:**

Pediatric Palliative Care (PPC) patients represent a highly heterogeneous population. Depending on the specific needs of the child and their family, PPC is provided at different levels, ranging from a basic PPC approach to highly specialized PPC in tertiary care centers. However, data on patients treated in specialized inpatient PPC units remain limited. This study aimed to further characterize this patient population, focusing on diagnostic spectrum, comorbidities, device dependency, admission patterns and mortality.

**Methods:**

A retrospective single-center study was conducted, including all patients admitted to an 8-bed specialized PPC unit at a tertiary, university-affiliated pediatric hospital in Germany between 2012 and 2023. Patient characteristics, underlying and secondary diagnoses based on the ICD-10-GM, device dependency, and admission patterns were analyzed descriptively.

**Results:**

*N* = 669 patients (mean age: 9.3; range: 0.03–33.5 years), cor-responding to *N* = 2,151 admissions, were analyzed. 47.7% of patients (*n* = 319) were readmitted at least once; the average number of inpatient stays was 3.2 (SD 4.7; range: 1–57) per patient. The mean length of stay was 12.6 days (SD 13.3; range 0–186) days. A total of 212 patients (31.7%) had diagnoses classified under chapter XVII (Congenital malformations, deformations, and chromosomal abnormalities), followed by 27.1% (*n* = 181) under chapter VI (Diseases of the nervous system) and 18.5% (*n* = 124) under chapter XVI (Certain conditions originating in the perinatal period). Nearly all patients (98.4%) had at least one secondary diagnosis, with a mean of 9.87 (range 1–23) secondary diagnoses per patient. A total of 74.6% of participants had at least one device. A total of 154 patients (23.0%) died within the observational period, and 57 (37.0%) were in the PPC unit. The mean distance between the patients' homes and the PPC unit was 69.7 (range 0–494.3) km.

**Conclusions:**

PPC patients exhibit profound multimorbidity, with the complexity of their healthcare needs necessitating specialized multidisciplinary care and a high level of clinical expertise within specialized PPC structures.

## 1 Introduction

An estimated 21 million children worldwide are in need of pediatric palliative care (PPC) (1). PPC addresses children with life-limiting or life-threatening conditions, as well as their families. In addition to its focus on symptom management, PPC aims to provide social and emotional support and enhance the quality of life of the entire unit of care, encompassing both the patient and their family. By addressing and alleviating physical, psychological, social, and spiritual suffering, PPC adopts a holistic care approach (2–4).

PPC patients constitute a highly heterogeneous patient group. They differ enormously in terms of their biological and developmental ages as well as underlying conditions (4–6). Many children suffer from rare or even undiagnosed neurological, genetic/congenital, neuromuscular, metabolic, or oncological conditions (1, 6–9). A substantial proportion of patients present with severe neurological impairment. They are characterized by profound functional limitations, an inability to communicate verbally, and an unpredictable and often prolonged disease trajectory. Many PPC patients require complex medical technologies and nursing care, such as home ventilation (10).

Additionally, PPC patients frequently experience multiple heterogeneous and interrelated symptoms. Common symptoms include respiratory problems, agitation, pain, seizures, and sleep disturbances (1, 11, 12). These underlying conditions may be stable but are also prone to limitations of muscle control, impaired sensation, and cognitive impairments, leading to a high risk of secondary health complications (13), such as severe lower respiratory tract infections (14). Consequently, PPC patients pose relevant challenges for medical, nursing, and psychosocial care.

Advances in medical treatments, technologies, and care structures have contributed to increasing survival rates among children with life-limiting conditions. Consequently, the prevalence of children and adolescents needing PPC has risen and is predicted to continue growing (15, 16). Fortunately, there has also been an increase in the number of PPC services in Europe in recent decades (17–19).

The first specialized PPC unit in Germany was established in 2010 at one of the largest university-affiliated pediatric hospitals in Germany. It provides highly specialized PPC by healthcare providers from various professional backgrounds. In Germany, children in need of PPC are cared for by multiple providers that each have a distinct lens for supporting patients and their families (20). In addition to general PPC, there are specialized PPC services, including children's and adolescents' hospices, specialized pediatric palliative home care teams, and specialized PPC units.

Specialized PPC units are considered if PPC patients can no longer be cared for at home – for example, due to crises, high symptom burden, or complex symptom clusters (12). Furthermore, inpatient stays on a specialized PPC unit may be particularly beneficial when new drugs (e.g., methadone or risperidone) are introduced, as they enable monitoring of efficacy, side effects, and potential drug interactions, which is especially relevant given the high prevalence of polypharmacy among PPC patients (6).

Despite their importance, there is limited systematic knowledge about the patient population treated in highly specialized inpatient PPC units. Existing literature has primarily focused on broader PPC populations or specific subgroups, leaving a gap in understanding the clinical complexity, diagnostic distribution, and healthcare utilization patterns within these highly specialized settings. Provision of care for PPC patients and their families can be improved by addressing these knowledge gaps regarding basic epidemiological data. Therefore, this study aimed to characterize the patient cohort treated over a period of 12 years in a specialized PPC unit. In detail the underlying PPC diagnoses, secondary diagnoses/symptoms, device dependency, admission/readmission patterns, place of death, and geographic catchment area are examined.

## 2 Patients and methods

### 2.1 Design

A retrospective single-center study was conducted to assess patient characteristics in an 8-bed specialized inpatient PPC unit at a tertiary university-affiliated hospital.

### 2.2 Setting

The specialized PPC inpatient unit was established in 2010 at a large university-affiliated pediatric hospital in Germany and provides highly specialized PPC for approximately 190 admissions annually, predominately for patients with neurological underlying diseases and their families (12). Up to eight patients can be accommodated in single rooms, and their families can stay in one of the seven family rooms or apartments. In addition to the unit's multiprofessional staff, healthcare providers from a variety of professional backgrounds collaborate in accordance with a bio-psycho-social-spiritual treatment approach (8) to ensure high quality care for the patients and their families. Therefore, in addition to the child's current state of health, the general wellbeing of the entire family is considered to achieve PPC treatment goals (e.g., reduced symptom burden, enhanced caregiver competencies, and improved quality of life) (12).

### 2.3 Participants

All patients admitted to the specialized PPC unit of the Children's and Adolescents' Hospital Datteln, Witten/Herdecke University, between January 2012 and December 2023 were eligible for inclusion. In line with the unit's scope, which serves children, adolescents, and selected young adults with ongoing pediatric palliative needs, the study included all admissions to the unit during the study period; therefore, no prespecified age limits were applied. Patients were excluded if they did not suffer from a life-limiting or life-threatening condition, as determined by identification from the medical records and reviewed according to established criteria for life-limiting and life-threatening conditions (3).

### 2.4 Data collection

Data were collected retrospectively. Data collection encompassed both analog (paper-based) and digital sources to ensure comprehensive capture of all relevant clinical information. Analog records, paper-based admission forms, and historical patient charts, were systematically digitized and verified for completeness and legibility prior to integration into the study database. Digital records were obtained from existing hospital information systems and electronic health records.

Data entry was performed by trained research personnel following a standardized protocol to minimize inter-rater variability. (A.K., D.G.). All analogue records were cross-checked against available digital sources where overlap existed, and discrepancies were resolved by a second reviewer. (D.V.) Following data entry, a systematic quality control process was conducted, including range checks, logical consistency checks, and duplicate record detection.

The underlying PPC diagnosis of each patient was identified from the medical records and reviewed according to established criteria for life-limiting and life-threatening conditions (3). In patients with multiple life-limiting conditions or unclear PPC diagnoses, cases were independently reviewed by two physicians (A.K, D.V) and subsequently adjudicated by consensus based on medical history and disease trajectory. Priority was assigned to the condition representing the earliest occurring life-limiting disease. For example, in patients with and hypoxic-ischemic encephalopathy in newborn (HIE) and chronic respiratory failure requiring long-term mechanical ventilation, HIE was defined as the primary PPC diagnosis, whereas respiratory insufficiency was considered a secondary consequence of the underlying disease. In cases of overlapping or clinically related diagnoses, diagnostic assignment was resolved by consensus after repeated chart review. Priority was given to the diagnosis associated with the greatest overall disease burden and the most substantial impact on the patient`s quality of life. Based on the International Statistical Classification Of Diseases and Related Health Problems, 10th Revision, German Modification (ICD-10-GM), the underlying life-limiting or life-threatening disease (PPC diagnosis) of each patient was assigned an ICD-10 code.

To enable structured analysis beyond the ICD-10 system, secondary diagnoses and symptoms were grouped into 16 categories. The grouping approach was developed by clinical consensus after evaluation of the available data: neurological, gastrointestinal, oral and maxillofacial, musculoskeletal, respiratory, pain-related, renal and urological, sensory, cardiovascular, haematological and oncological, dermatological, endocrine and metabolic, electrolyte and fluid imbalances, mental, congenital and neonatal, and gynaecological (Supplementary Table S2). Multiple categories could be assigned to a patient, and several secondary diagnoses could occur within the same category.

Missing or incomplete data on general patient characteristics and underlying PPC diagnoses were retrospectively completed through review of all available medical records, including retrieval of archived records.

### 2.5 Analyses

All analyses were performed using IBM SPSS Statistics version 29. Data were analysed descriptively. All analyses, except for admissions per year, were analysed at the patient level. Data from all admissions were included in the analysis of annual admission rate. Regarding the analysis of secondary diagnoses, patients without documented secondary diagnoses were included in the calculation of chapter-specific means.

## 3 Results

Over the 12-year period (2012–2023), a total of *n* = 702 patients were admitted to the specialized PPC unit, accounting for *n* = 2,184 admissions. According to the exclusion criteria, *n* = 33 patients (4.3%; corresponding to 1.5% of admissions) were excluded because they did not suffer from a life-limiting or life-threatening disease.

Thus, data from *n* = 669 patients (comprising *n* = 2,151 admissions) were included in the analyses.

### 3.1 Patients characteristics

Of the *n* = 669 patients admitted to the specialized PPC unit, 45.7% (*n* = 306) were female. At the first documented admission, 10.2% (*n* = 68) of patients were aged < 1 year, whereas 8.2% (*n* = 55) were older than 18 years. Nearly half of the patients (47.7%; *n* = 319) were readmitted at least once during the study period (Table 1).

**Table 1:** Patients' characteristics at the patient level (*n* = 669) and admission level (*n* = 2,151).

Characteristics		N (%)	Mean (SD)	Median (IQR)	Range
		Patient level			
Sex (female)		306 (45.7)			
Readmissions during the study period		319 (47.7)			
Average number of inpatient stays per patient			3.2 (4.7)	1 (2)	1–57
Deceased during observation period		154 (23.0)			
Place of death (*n* = 154)					
Specialized PPC unit		57 (37.0)			
Hospital		44 (28.6)			
Home		41 (26.6)			
Hospice		5 (3.2)			
Unknown		7 (4.4)			
Distance between PPC unit and home			69.7 (94.9)	29.8 (58.3)	0- 494.3
< 10 km		58 (8.7)			
11–50 km		379 (56.7)			
51–100 km		111 (16.6)			
101–150km		32 (4.8)			
151–200km		24 (3.6)			
201–250km		11 (1.6)			
>250		54 (8.1)			
		Admission level			
Age			9.3 (6.6)	8.2 (11.3)	0.03- 33.5
Length of stay (days)			12.6 (13.3)	9 (13)	0- 186

**Figure 1:**
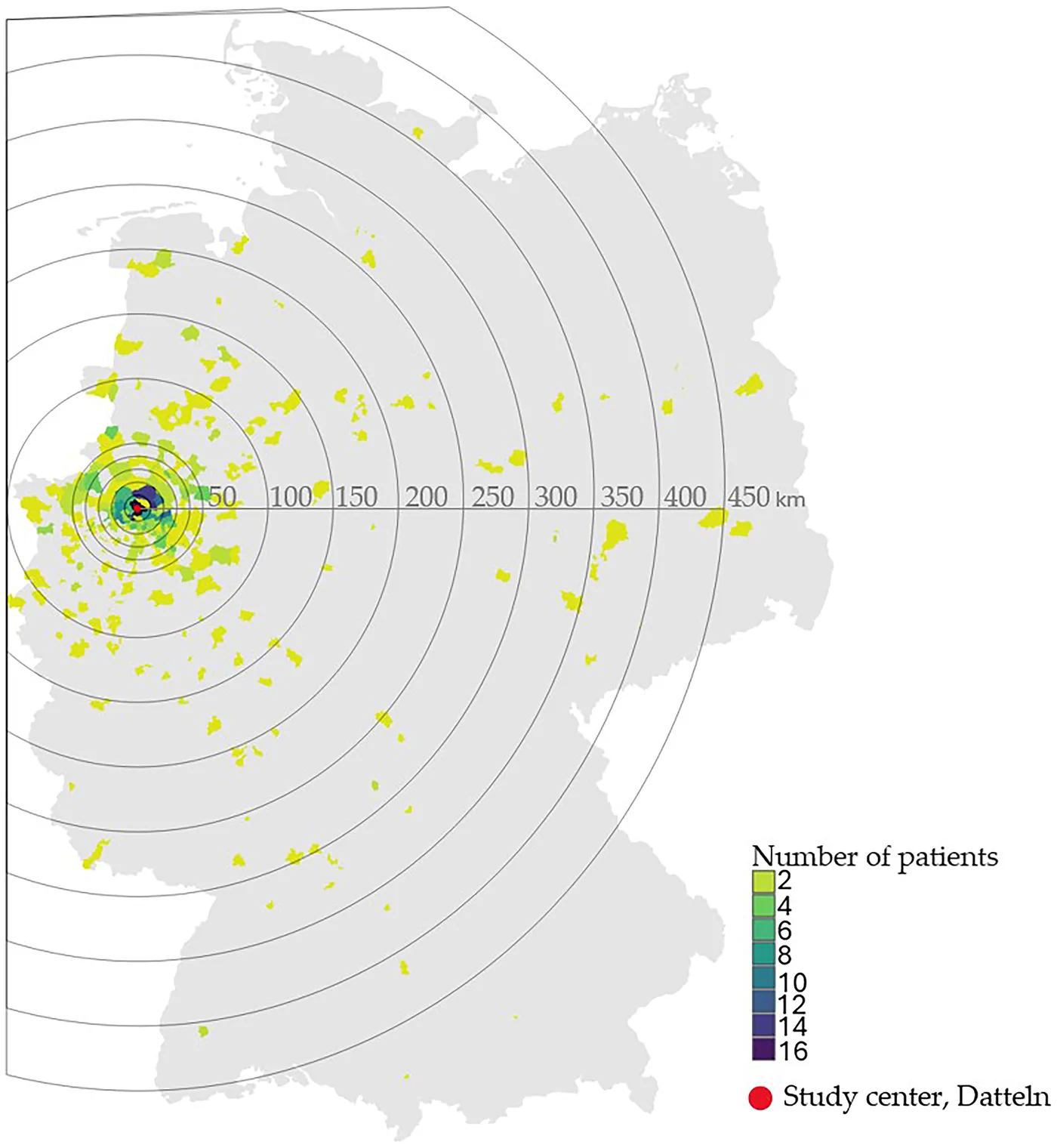
Distance between specialized PPC unit and home (*n* = 667; two patients from Luxembourg were not included in the figure).

The mean distance between the PPC unit and the patients' homes was 69.7 km (SD: 94.9, range: 0–494.3). Most patients (*n* = 437; 65.4%) lived within 50 km of the specialized PPC unit (Figure 1). Overall, 80% of patients (*n* = 535) resided within a catchment area of 92 km.

### 3.2 Underlying PPC diagnosis

**Figure 2:**
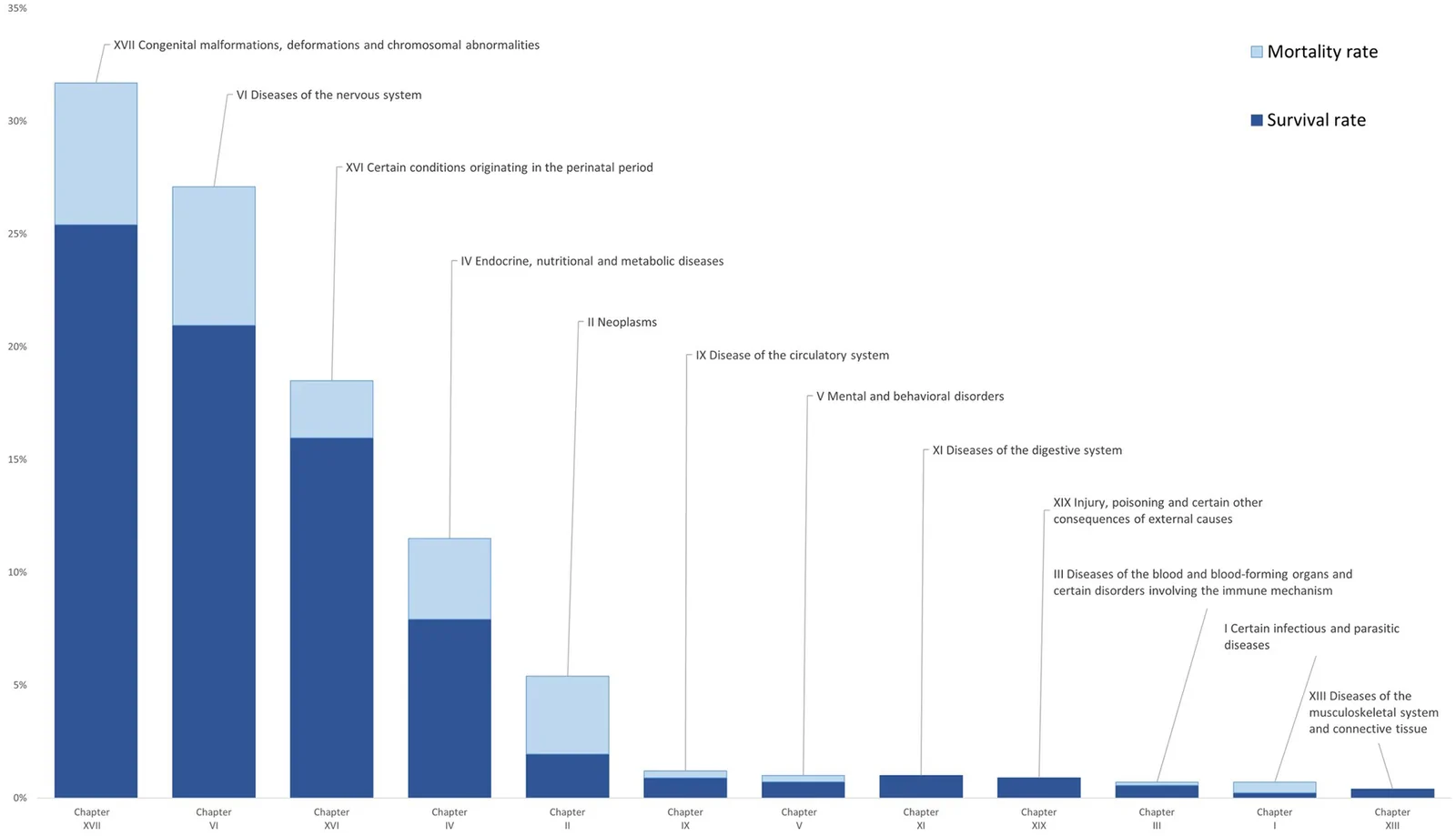
Mortality and survival rates by patient PPC diagnosis, stratified by respective ICD-10 chapter.

Overall, the patients showed a broad range of underlying PPC diagnoses distributed across 12 chapters of the ICD-10-GM classification system (Figure 2). The majority of PPC diagnoses (94.2%) were distributed across five ICD-10-GM chapters.

The most frequent diagnoses (*n* = 212, 31.7%) were assigned to chapter XVII (Congenital malformations, deformations and chromosomal abnormalities), followed by 27.1% (*n* = 181) assigned to chapter VI (Diseases of the nervous system), and 18.5% (*n* = 124) to chapter XVI (Certain conditions originating in the perinatal period).

**Figure 3:**
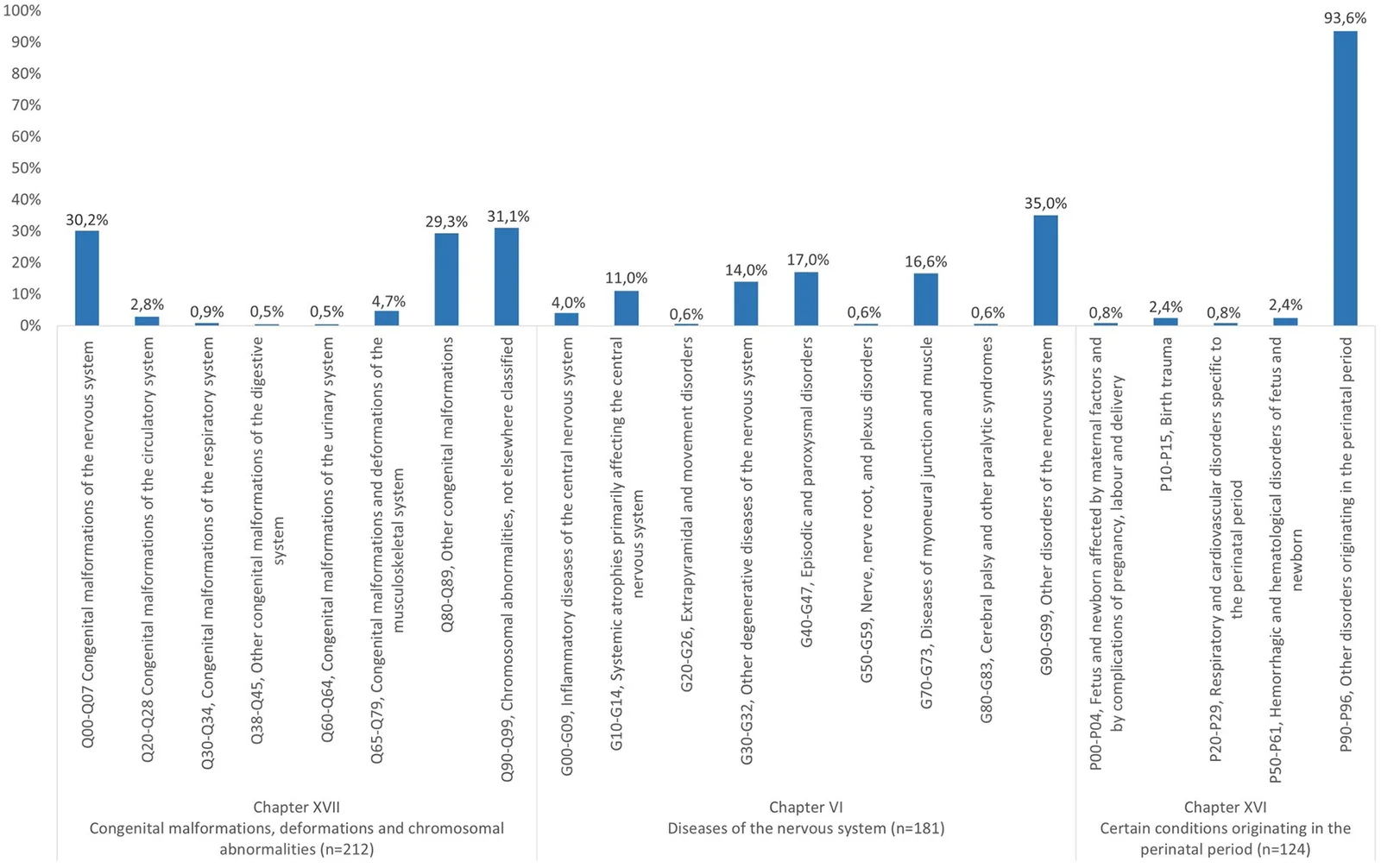
Overview of the associated blocks of the three most frequent ICD-10 chapters.

A more detailed overview of the associated blocks of the three most frequent ICD-10-GM chapters (XVII, VI and XVI) is demonstrated in Figure 3.

At the level of individual ICD-10-GM codes, the most frequent diagnosis, P91.6 (hypoxic-ischemic encephalopathy in newborn), accounted for 11.4% of cases (*n* = 76). Among the remaining codes, G93.1 (anoxic brain damage, not elsewhere classified) and P96.8 (other specified conditions originating in the perinatal period) were the next most frequent, each comprising 4.6% (*n* = 31), followed by G93.4 (Encephalopathy, unspecified) at 4.0% (*n* = 27). A detailed overview of PPC diagnosis frequencies is provided in Supplementary Table S1.

### 3.3 Secondary diagnosis

In addition to the underlying PPC diagnosis, 98.4% (*n* = 658) of patients had at least one secondary diagnosis or related symptom. On average, these 658 patients had 9.87 (SD 4.36, range: 1–23) secondary diagnoses. Table 2 presents the average number of secondary diagnoses stratified by underlying PPC diagnosis.

**Table 2:** Average number of secondary diagnoses per patient in relation to the underlying PPC diagnosis.

Underlying PPC diagnosis			Secondary diagnoses	
ICD-10 Chapter		Patients (n)	Mean (SD)	Range
XVII	Congenital malformations, deformations and chromosomal abnormalities	212	9.9 (4.4)	0–21
VI	Diseases of the nervous system	181	9.0 (4.2)	0–23
XVI	Certain conditions originating in the perinatal period	124	11.52 (4.6)	0–23
IV	Endocrine, nutritional and metabolic diseases	77	10.2 (4.0)	0–16
II	Neoplasms	36	6.4 (4.0)	0–19
IX	Disease of the circulatory system	8	10.1 (3.6)	3–14
V	Mental and behavioral disorders	7	8.6 (4.7)	1–16
XI	Disease of the digestive system	7	4.1 (1.7)	1–6
XIX	Injury, poisoning and certain other consequences of external causes	6	8.5 (4.9)	3–17
III	Diseases of the blood and blood-forming organs and certain disorders involving the immune mechanism	5	8.2 (6.9)	0–18
I	Certain infectious and parasitic diseases	3	12 (4.6)	7–16
XIII	Diseases of the musculoskeletal system and connective tissue	3	8 (7.9)	2–17

**Figure 4:**
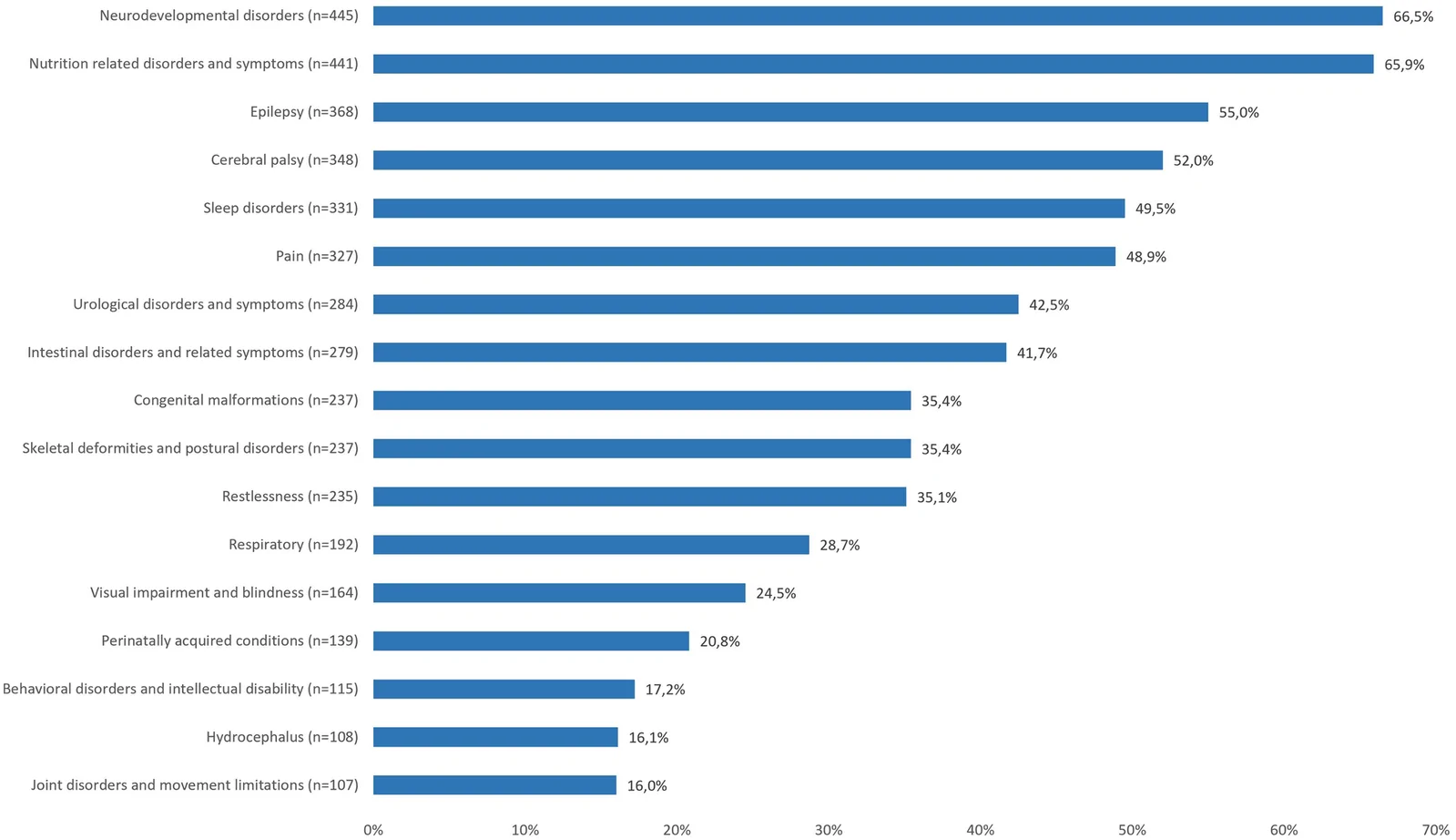
Most frequent secondary diagnoses and symptoms (restricted to those with 100 or more cases).

Neurological secondary diagnoses or related symptoms were present in 84.2% (*n* = 563) of patients. Mental health-related diagnoses were documented in 78.8% (*n* = 527); most commonly neurodevelopmental disorders (66.5%) and restlessness (35.1%). Gastrointestinal diagnoses affected 75.9% (*n* = 508) of patients, predominantly comprising nutrition-related disorders and symptoms (65.9%; *n* = 441). A detailed overview of the most frequent secondary diagnoses and symptoms is presented in Figure 4.

### 3.4 Devices and opioid therapy

Overall, 74.6% (*n* = 499) of patients had at least one medical device. Percutaneous endoscopic gastrostomy (PEG) was present in 50.2% (*n* = 336), cerebrospinal fluid drainage in 9.6% (*n* = 64), and 8.1% (*n* = 54) had a tracheostoma. Respiratory support via mechanical ventilation was required in 8.7% (*n* = 58), and 39.0% (*n* = 261) were wheelchair dependent.

During the study period, *n* = 144 (21.5%) received opioid therapy. Among patients who died during the study period, 46.8% (*n* = 72) had received opioids.

### 3.5 Admission

Over the 12-year study period, a total of *n* = 2,151 admissions were recorded. Readmissions accounted for 69.9% (*n* = 1,482) of all admissions.

Patients had a mean of 3.2 admissions during the study period, with an average length of stay of 12.6 days (Table 1). Patients living within 10 km of the PPC unit (*n* = 58) had a mean of 4.3 admissions (SD = 5.9), compared to 1.7 admissions (SD = 1.7) among patients living more than 250 km away (*n* = 54).

**Figure 5:**
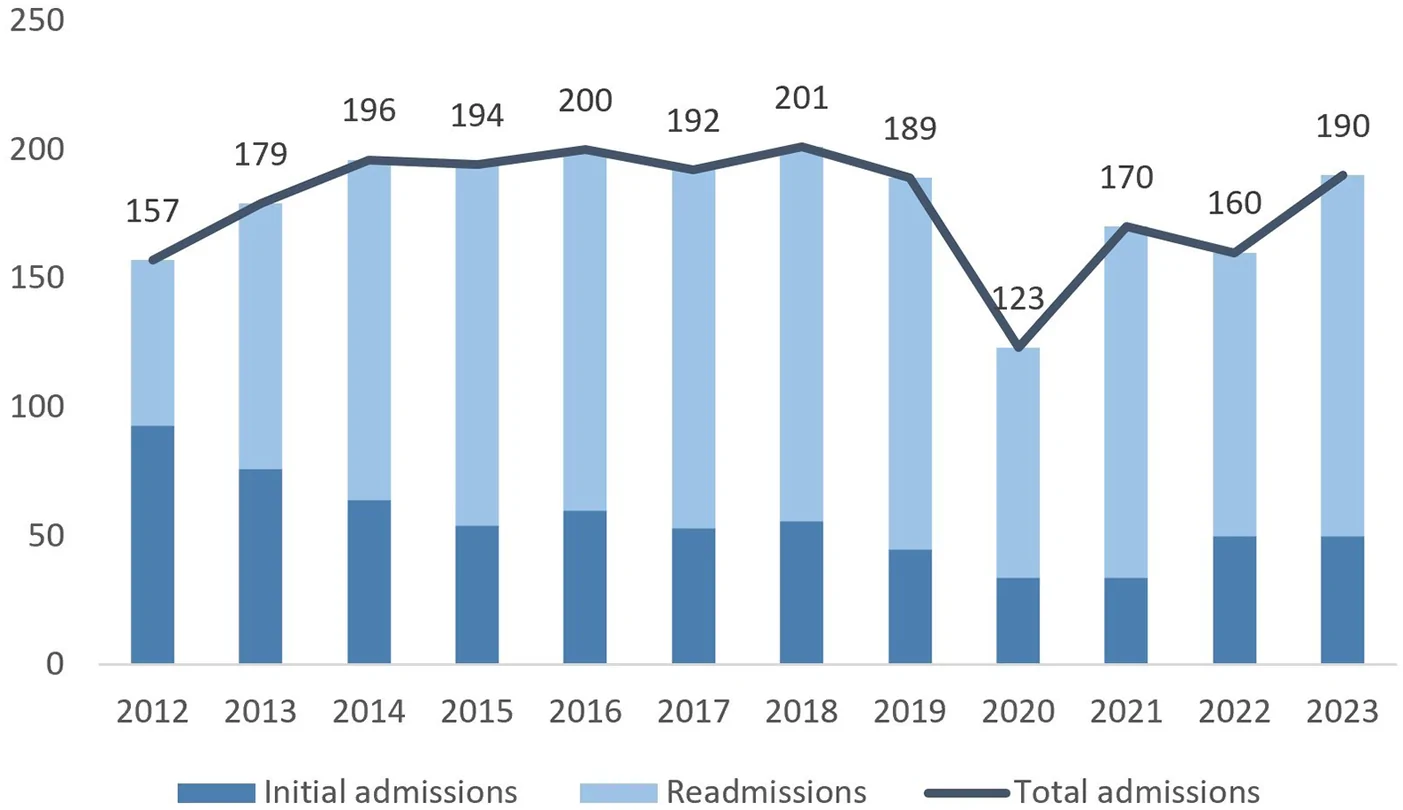
Number of (re)admissions to the PPC unit per year.

Figure 5 shows a relatively stable number of approximately 190 admissions per year, with a decrease during the pandemic years 2020–2022. The proportion of first admissions declined from 59.2% (*n* = 93) in 2012 to 26.3% (*n* = 50) in 2023.

The distribution of ICD-10 chapters of PPC diagnoses associated with admissions is presented in Table 3. Chapter VI, Diseases of the nervous system, accounted for the largest proportion of all admissions (30.3%) and readmissions (31.8%).

**Table 3:** Five most frequent ICD-10 chapters among the *n* = 2,151 admissions.

ICD-10 Chapter	Diagnosis category		Patients with	
		Admissions n (%)	Single admission n (%)	>1 admission n (%)
VI	Diseases of the nervous system	652 (30.3)	181 (27.2)	471 (31.8)
XVII	Congenital malformations, deformations and chromosomal abnormalities	629 (29.2)	212 (31.7)	417 (28.1)
XVI	Certain conditions originating in the perinatal period	402 (18.7)	124 (18.5)	278 (18.8)
IV	Endocrine, nutritional and metabolic diseases	282 (13.1)	77 (11.5)	205 (13.8)
II	Neoplasms	69 (3.2)	36 (5.4)	33 (2.2)

## 4 Discussion

This study intended to characterize patients treated at a specialized inpatient PPC unit in Germany over a period of 12 years. Overall, the findings demonstrate that patients treated in this setting represent a highly complex and heterogeneous population with substantial multimorbidity, frequent device dependency, and recurrent hospitalizations. The broad age distribution within the cohort highlights the wide spectrum of care needs and the associated challenges for healthcare providers. The inclusion of patients older than 18 years underscores the growing need for structured transition pathways and appropriate adult care facilities for childhood-onset life-limiting conditions, as survival into adulthood is increasingly common (5, 21). Particularly in patients with highly complex diseases and ongoing intensive medical care needs, transition to adult care is often delayed, resulting in prolonged care within established pediatric care structures.

The patients showed a broad range of different underlying PPC diagnoses distributed across 12 chapters of the ICD-10-GM classification system. Most (31.7%) were assigned to chapter XVII, Congenital malformations, deformations and chromosomal abnormalities. Analysis of individual ICD-10-GM codes revealed that P91.6, hypoxic-ischemic encephalopathy in newborn, was the most frequent code. Nearly all patients (98.4%) had at least one secondary diagnosis, and 74.6% had at least one device. Moreover, the majority of patients were ad-mitted multiple times during the study period.

Analysis of PPC-diagnoses based on ICD-10-GM classification showed a pronounced concentration within a small subset of chapters; five of the twelve chapters accounted for 94.2% of all underlying PPC diagnoses. This distribution is consistent with the existing literature (22). Subsequent analyses at the level of ICD-10-GM blocks and individual codes revealed a much broader distribution across blocks and specific diagnostic codes. Moreover, these data highlight the heterogeneity of patients cared for in PPC (4, 5). Compared to specialized adult palliative care units in Germany (23, 24), results clearly highlight the differences among the patients to be treated. In addition to the wide age range covered by PPC, the range of medical specialties is also considerably broader than in specialized adult palliative care. Furthermore, specialized PPC patients present additional challenges, such as rapidly changing symptoms, a lack of approved necessary medications, and prolonged periods of stagnation in the pre-final phase that may last weeks or even months (20). This underlines the necessity of interdisciplinary care structures within PPC as well as the important need for a well-trained team to face all these challenges.

Fortunately, medical and technical advances increase life expectancy for children with PPC needs, especially patients with underlying PPC diagnoses affecting the nervous system (chapters XVII, VI, and XVI). Therefore, it can be assumed that the demand for technical devices grew for patients admitted to the specialized PPC unit. Because device dependency was assessed at the patient level and not at each individual admission, this cannot be verified. Additionally, technical progress has led to the establishment of specialized PPC units that increasingly resemble pediatric intensive care units (PICU). This also requires a highly trained team within a specialized PPC unit and represents another distinction compared to adult Palliative Care (PC) units.

Ongoing advances in medical treatments and technological developments, especially in relation to medical devices used by children requiring PPC, have contributed to increased life expectancy and growing demand for specialized PPC units. Currently, there are three specialized PPC units in Germany. Given the affected population of children and the prevalence estimates for the coming years reported by Kubek et al. (16), this provision is far from sufficient. In comparison, there are currently 372 PC units for adults in Germany; nearly each German citizen is able to reach a PC unit within 30 min by car (25). Results demonstrate 80% of patients admitted to the specialized PPC unit reside within a catchment area of 92 km, which is considerably more than a 30-minute car drive.

Consistent with the literature (26–28), most of the deceased children had an underlying PPC diagnosis under chapters XVII (congenital malformations, deformations and chromosomal abnormalities) and VI (diseases of the nervous system), followed by chapters IV (endocrine, nutritional and metabolic diseases) and II (neoplasms). The highest mortality rates occurred in patients with an underlying PPC diagnosis under chapter II, neoplasms.

A high degree of multimorbidity was observed in the secondary diagnoses, alongside frequent dependence on various medical devices and complex medical technologies. Consistent with the existing literature (5, 22), neurological conditions were the most prevalent secondary diagnoses. These findings demonstrate the necessity of specialized, multidisciplinary, and interdisciplinary PPC (4), and further underscore the breadth of knowledge and expertise required among PPC professionals to provide care for this heterogeneous and complex patient group.

Overall, the results indicate high healthcare utilization during the study period. A readmission rate of 47.7% and an average of 3.22 admissions per patient indicate a considerable disease burden and recurrent clinical decompensation. Regarding the corresponding ICD-10-GM chapters, analyses at the admission level show the same pattern as was observed at the patient level.

### 4.1 Limitations

This study has some limitations. First, it was conducted in a single specialized PPC unit in Germany, which may limit the generalizability of the findings to other settings. However, the setting reflects a typical specialized inpatient unit. Future studies should aim to replicate these findings in multicenter or international contexts.

Second, the reasons for admission to the specialized PPC unit, the intensity of care as well as symptom severity and patient-/family- reported-outcomes were not assessed in this study but represent important aspects for future research.

Third, secondary diagnoses and device dependency were assessed at the patient level but not longitudinally across subsequent patient admissions. Therefore, trends in terms of secondary diagnoses and dependence on medical devices or health technologies could not be identified. Additionally, the completeness of secondary diagnoses could not be reassessed retrospectively and may therefore be subject to underreporting.

Finally, classifying the patients based on the five Categories of Life-Limiting and Life-Threatening Conditions (4) might have ensured greater international comparability.

## 5 Conclusions

These results elucidate that patients of a highly specialized PPC unit show a broad range of underlying PPC diagnoses, high secondary diagnosis burden, frequent device dependency, and recurrent hospitalizations, which indicate a considerable disease burden and recurrent clinical decompensation. Therefore, the need for i) widespread specialized PPC structures, ii) well-trained specialized PPC teams, and iii) support for all family members is warranted. To confirm generalizability multicenter studies would be useful.

## Data Availability

The datasets presented in this article are not readily available because the dataset contains sensitive patient information. Due to ethical, legal, and data protection requirements, the data cannot be made publicly available. Requests to access the datasets should be directed to Pia Schmidt (MScN, PhD), p.schmidt@kinderpalliativzentrum.de.
