# Supplementary material for: 12 years of specialized pediatric palliative inpatient care: description of patients' characteristics
Source: Front Pediatr. 2026 Aug 28;14:1915934. doi: 10.3389/fped.2026.1915934 (PMC13562009; doi:10.3389/fped.2026.1915934)
Supplement: Supplementary file 1 [file Datasheet1.zip › Table S2. Secondary diagnoses and related symptoms with corresponding ICD-10-GM codes.pdf]

Table S2. Secondary diagnoses and related symptoms with corresponding ICD-10-GM codes (N=669).

Category	Subcategory	ICD-10 Code	N (%)
Neurological			563 (84.2)
	Ataxia		8 (1.2)
		R27.0, R27.8	
	Cerebral palsy		348 (52.0)
		G80.0, G80.1, G80.2, G80.3, G80.4, G80.8, G80.9, G81.1, G81.9, G82.19, G82.22, G82.23, G82.29, G82.42, G82.43, G82.49, G82.53, G82.59, G82.64, G82.69, G83.0, G83.8	
	Cerebrovascular disorders		12 (1.8)
		G45.1, I61.0, I61.4, I62.9, I63.9, I67.88, I69.0, I69.2, I69.3, I69.8, S06.8, T90.5	
	Degenerative and neurodegenerative disorders		63 (9.4)
		F02.8, G12.2, G31.0, G31.81, G31.88, G31.9, G32.8, G37.8, G37.9, G93.0, G93.8	
	Epilepsy		368 (55.0)
		G40.0, G40.08, G40.09, G40.1, G40.2, G40.3, G40.4, G40.5, G40.6, G40.8, G40.9, G41.0, G41.8, G41.9	
	Extrapyramidal disorders		48 (7.2)
		G24.8, G24.9, G25.3, G25.88, G25.9	
	Hydrocephalus		108 (16.1)
		G91.0, G91.1, G91.29, G91.8, G91.9, Q03.9, G05.1, Q05.2, Q05.4	
	Inflammatory disorders of the central nervous system		6 (0.9)
		G04.2, G04.9, G09, G96.1	
	Other convulsions		22 (3.3)
		F44.5, P90, R25.1, R25.2, R56.0, R56.8	
	Other neurological disorders and related symptoms		59 (8.8)
		G24.4, G25.2, G50.0, G50.9, G51.0, G55.1, G60.0, G62.0, G62.9, G81.0, G82.33, G90.9, G93.2, G93.6, G93.80, G93.88, G93.9, G95.0, G95.83, G95.88, G97.88, R29.8, R40.2, R42, R46.1, R50.88, R50.9, R55, R83, R94.0, S14.11, T75.1, T91.3, T98.1	
	Sleep disorders		331 (49.5)
		F51.4, F51.9, G47.0, G47.2, G47.30, G47.31, G47.32, G47.38, G47.8, G47.9, R40.0	
Gastrointestinal			508 (75.9)
	Esophageal disorders		87 (13.0)
		I85.0, I85.9, K20.8, K21.0, K21.9, K22.2, K22.4, K22.7	
	Intestinal disorders and related symptoms		279 (41.7)
		K42.9, K44.9, K56.0, K56.7, K59.01, K59.09, K59.1, K59.2, K59.3, K59.8, K59.9, K60.1, K60.2, K63.1, K63.2, K92.0, K92.1, K92.2, R14, R15, T85.5, T85.51	
	Stomach and duodenum disorders and related symptoms		18 (2.7)
		I86.4, K29.0, K29.5, K29.6, K31.1, K31.88, K31.9, K91.0, R11	
	Liver and gallbladder disorders		9 (1.3)
		K71.6, K74.0, K74.6, K75.8, K76.0, K76.9, K80.20	
	Nutrition related disorders and symptoms		441 (65.9)
		E41, E43, E44.0, E44.1, E45, E46, E50.0, E50.9, E53.8, E53.9, E60, E61.2, E63, E63.9, K90.4, K90.8, K91.2, K93.8, R13, R13.0, R13.1, R13.9, R63.0, R63.3, R63.4, R63.5, R64	
	Pancreatic disorders		3 (0.4)
		K85.01, K86.2, K86.3	
Oral and maxillofacial			22 (3.3)

	K02.8, K02.9, K05.1, K06.1, K07.0, K07.1, K11.2, K11.7	
Musculoskeletal		296 (44.2)
	Bones and joints disorders and related symptoms	72 (10.8)
	M08.00, M08.20, M08.90, M13.96, M13.99, M35.9, M80.25, M81, M81.2, M81.20, M81.21, M81.	
	Joint disorders and movement limitations	107 (16.0)
	M22.0, M23.99, M24.30, M24.34, M24.35, M24.36, M24.40, M24.45, M24.49, M25.35, M25.45, T98.3, T98.3	
	Muscle and nerve disorders and related symptoms	10 (1.5)
	G70.8, G72.88, G72.9, G73.7, M62.40, M62.50, M62.89, R25.3, R26.8	
	Skeletal deformities and postural disorders	237 (35.4)
	M21.05, M21.07, M21.57, M21.6, M21.62, M21.63, M21.86, M24.50, M24.52, M24.53, M24.55, M24.56, M24.59, M25.50, M40.20, M40.29, M40.46, M41.09, M41.19, M41.20, M41.29, M41.40, M41.45, M41.49, M41.53, M41.54, M41.55, M41.56, M41.59, M41.80, M41.84, M41.85, M41.89, M41.9, M41.90, M41.94, M41.95, M41.96, M41.99, M43.90, M48.04, M48.54, M53.0, M95.2, M95.4, R29.3	
Respiratory		192 (28.7)
	J96.00, J44.19, J38.00, J39.80, J39.88, J44.89, J45.99, J80, J86.0, J96.19, J96.99, J98.0, J35.2, J38.6, J41.1, J44.09, J45.9, R06.88, J96.10, J96.11, J98.4, J98.6, J98.12, R06.0, R06.1, R09.2, I27.2	
Pain-related		335 (50.1)
	Headaches	21 (3.1)
	G43.0, G43.1, G43.8, G43.9, G44.2, R51	
	Pain	327 (48.9)
	F45.1, F45.41, F45.8, G54.6, M25.55, M54.18, M54.5, M54.90, M54.99, M79.10, M79.19, M79.60, M79.65, M79.67, R10.3, R10.4, R52, R52.0, R52.1, R52.2, R52.9	
Renal and urological		292 (43.6)
	Renal disorders and symptoms	17 (2.5)
	N08.5, N11.9, N16.3, N17.89, N18.5, N18.80, N18.89, N18.9, N20.0, N28.1, N28.88, Q60.2, Q61.8, Z94.0, Z99.2	
	Urological disorders and symptoms	284 (42.5)
	G95.80, G95.81, N13.7, N28.88, N31.2, N31.88, N31.9, N32, N32.8, N32.9, N39.0, N47, Q62.7, R32, R33, R35.1, R39.1	
Sensory		217 (32.4)
	Hearing disorders and related symptoms	81 (12.1)
	H65.2, H66.9, H90.0, H90.3, H90.5, H90.6, H91.0, H91.1, H91.8, H91.9, H93.2, R44.8, Z01.1	
	Visual impairment and blindness	164 (24.5)
	H02.2, H02.4, H02.5, H10.4, H17.8, H18.8, H26.9, H35.0, H35.39, H40.9, H42.0, H42.8, H47.2, H48.0, H49.0, H49.2, H49.8, H50.0, H50.1, H50.5, H51.9, H52.0, H52.1, H52.2, H53.0, H53.2, H53.4, H53.8, H53.9, H54.0, H54.1, H54.4, H54.9, H55, H57.0, Q15.0	
Cardiovascular		90 (13.5)
	Functional and intervention-related disorders and symptoms	30 (4.5)
	I10.0, I12.01, I13.0, I15.90, I45.1, I45.8, I46.0, I46.9, I47.1, I49.3, I49.4, I49.8, I49.9, I50.01, I50.9, I95.9, R00.0, R00.1, T82.5	
	Structural disorders and related symptoms	65 (9.7)
	I07.1, I08.0, I27.0, I27.28, I34.0, I35.0, I35.1, I36.1, I42.0, I42.2, I42.88, I42.9, I51.4, I51.7, I73.9, Z94.1	
Hematological and oncological		131 (19.6)

Anemia		71 (10.6)
	D50.0, D50.8, D50.9, D55.0, D56.0, D56.1, D56.9, D58.0, D61.10, D62, D63.0, D63.8, D64.1, D64.8, D64.9	
Benign neoplasms		13 (1.9)
	D15.1, D18.02, D18.03, D18.05, D18.08, D22.5, D30.0, D33.2, D35.2, D36.1, D36.7, D36.9, D44.8	
Coagulopathies and thrombocytopenia		32 (4.8)
	D47.3, D66, D68, D68.0, D68.09, D68.22, D68.23, D68.4, D68.5, D68.8, D68.9, D69.1, D69.58, D69.59, D69.61, R58	
Malignant neoplasms		17 (2.5)
	C64, C74.1, C74.9, C76.2, C77.3, C77.8, C78.0, C78.6, C78.7, C79.0, C79.3, C79.4, C79.5, C79.88, C79.9, C80.9, C83.7	
Neutropenia		7 (1.0)
	D70.10, D70.12, D70.18, D70.7	
Other hematological disorders and symptoms		25 (3.7)
	A41.9, B34.0, B50.0, D76.1, D80.1, D80.9, D90, D90, I82.3, I82.80, I82.9, I98.1, R16.1, R59.1, T86.01, Z86.2, Z88.3, Z94.80, Z94.81, Z94.88, Z94.9	
Dermatological		51 (7.6)
Decubitus		16 (2.4)
	L89.0, L89.00, L89.04, L89.05, L89.06, L89.08, L89.09, L89.10, L89.14, L89.17, L89.18, L89.25	
Other dermatological disorders and symptoms		35 (5.2)
	I89.0, I89.8, L04.0, L27.0, L27.1, L29.0, L29.8, L29.9, L30.3, L74.4, L92.8, L92.9, L93.0, L98.4, Q80.9, Q81.9, Q84.8, Q85.8, R02, R20.8, R21, R23.3, R60.0, R61.1, R61.9, Z94.5	
Endocrine and metabolic		120 (17.9)
Endocrine disorders		81 (12.1)
	E03.1, E03.8, E03.9, E06.3, E10.61, E10.90, E21.1, E23.0, E23.2, E24.2, E25.00, E27.2, E27.4, E27.8, E55.9, E89.0	
Growth and pubertal development disorders		25 (3.7)
	E30.0, E30.1, E34.3, E34.4	
Metabolic disorders		20 (3.0)
	E66.29, E66.94, E66.99, E70.2, E71.3, E72.1, E73.9, E74.8, E75.2, E77.8, E83.38, E88.8, E88.9	
Electrolyte and fluid imbalances		12 (1.8)
	E16.2, E83.58, E86, E87.3, E87.6, R17, R29.0, R73.9, R74.8	
Mental		527 (78.8)
Behavioral disorders and intellectual disability		115 (17.2)
	F06.7, F06.9, F07.2, F11.2, F13.2, F62.80, F63.3, F70.1, F70.9, F71.0, F71.1, F71.8, F72.0, F72.1, F72.9, F73.0, F73.1, F73.9, F74.0, F79.0, F79.1, F79.8, F79.9, F84.0, F84.1, F91.8, F92.8, F93.0, F93.8, F93.9, F98.8	
Child abuse		6 (0.9)
	T74.0, T74.1, T74.8	
Depressive and anxiety disorders		49 (7.3)
	F32.1, F32.9, F40.2, F40.9, F41.0, F41.1, F41.2, F41.8, F41.9, F43.0, F43.1, F43.2, F43.8, F45.2, F54, R45.3, R53	
Neurodevelopmental disorders		445 (66.5)
	F80.1, F80.28, F80.8, F80.9, F81.8, F81.9, F82, F82.0, F82.1, F82.2, F82.9, F83, F84.8, F84.9, F88, F89, F89, R47.0, R47.1, R47.8, R62.0, R62.8, R62.9	
Eating disorders and substance abuse		47 (7.0)

		F11.3, F19.1, F19.2, F19.3, F50.8, F50.9, F55.2, T50.9, Y57.9	
	Restlessness		235 (35.1)
		R45.1	
Congenital and neonatal			323 (48.3)
	Chromosomal abnormalities		16 (2.4)
		Q92.8, Q93.5, Q93.9, Q99.8, Q99.9	
	Congenital malformations		237 (35.4)
		G93.4, Q02, Q03.0, Q03.1, Q04.0, Q04.2, Q04.3, Q04.5, Q04.6, Q04.8, Q04.9, Q05.8, Q05.9, Q06.1, Q07.0, Q11.1, Q11.2, Q13.2, Q13.3, Q13.8, Q14.1, Q15.8, Q16.9, Q18.9, Q20.1, Q20.3, Q20.4, Q21.0, Q21.1, Q21.2, Q21.4, Q22.1, Q22.8, Q22.9, Q23.1, Q23.3, Q23.8, Q24.8, Q24.9, Q25.0, Q25.1, Q25.5, Q25.6, Q25.7, Q25.8, Q26.1, Q27.9, Q28.88, Q30.0, Q31.1, Q31.5, Q31.8, Q32.0, Q32.1, Q32.2, Q32.3, Q33.6, Q33.8, Q34.8, Q35.5, Q35.9, Q37.9, Q38.2, Q38.5, Q39.3, Q40.0, Q42.3, Q43.1, Q43.3, Q45.3, Q53.2, Q53.9, Q54.9, Q60.0, Q61.4, Q63.0, Q63.1, Q64.7, Q65.0, Q65.1, Q65.8, Q66.0, Q66.3, Q66.4, Q66.6, Q66.7, Q66.8, Q67.3, Q67.6, Q67.7, Q67.8, Q69.9, Q71.9, Q72.9, Q74.0, Q74.3, Q74.8, Q75.0, Q75.2, Q75.3, Q75.8, Q76.3, Q76.4, Q76.9, Q77.3, Q77.4, Q79.3, Q79.8, Q79.9, Q86.0, Q86.88, Q87.0, Q87.1, Q87.5, Q87.8, Q89.0, Q89.2, Q89.8, Q89.9	
	Perinatally acquired conditions		139 (20.8)
		G93.0, G93.1, I69.1, P01.1, P07.00, P07.01, P07.11, P07.12, P07.2, P07.3, P21.1, P21.9, P27.1, P27.8, P28.4, P29.1, P35.1, P37.2, P52.0, P71.2, P76.9, P77, P90, P91.2, P91.6, P91.7, P91.9, P94.2, P96.0, P96.8, Z87.6	
Gynecological			3 (0.4)
		N92.5, N94.6, N94.9	
