# Supplementary material for: 12 years of specialized pediatric palliative inpatient care: description of patients' characteristics
Source: Front Pediatr. 2026 Aug 28;14:1915934. doi: 10.3389/fped.2026.1915934 (PMC13562009; doi:10.3389/fped.2026.1915934)
Supplement: Supplementary file 1 [file Datasheet1.zip › Table S1. PPC diagnosis frequencies across ICD-10-GM classification system (N=669)..pdf]

Table S1. PPC diagnosis frequencies across ICD-10-GM classification system (N=669).

Chapter	Block	ICD-10-GM code	N (%)
I Certain infectious and parasitic diseases			3 (0.4)
	A80-A89, Viral infections of the central nervous system		2 (0.3)
		A81.1, Subacute sclerosing panencephalitis	1 (0.1)
		A81.2, Progressive multifocal leukoencephalopathy	1 (0.1)
	B90-B94, Sequelae of infectious and parasitic diseases		1 (0.1)
		B94.9, Sequelae of unspecified infectious or parasitic disease	1 (0.1)
II Neoplasms			36 (5.4)
	C15-C26, Malignant neoplasms of digestive organs		5 (0.7)
		C22.0, Liver cell carcinoma	3 (0.4)
		C22.4, Other and unspecified malignant neoplasms of liver	1 (0.1)
		C22.7, Other specified carcinomas of liver	1 (0.1)
	C40-C41, Malignant neoplasms of bone and articular cartilage		3 (0.4)
		C40.0, Malignant neoplasm of bone and articular cartilage of limbs	3 (0.4)
	C45-C49, Malignant neoplasms of mesothelial and soft tissue		6 (0.9)
		C47.8, Malignant neoplasm of overlapping sites of peripheral nerves and autonomic nervous system	1 (0.1)
		C49.3, Malignant neoplasm of other connective and soft tissue of thorax	1 (0.1)
		C49.4, Malignant neoplasm of other connective and soft tissue of abdomen	2 (0.3)
		C49.5, Malignant neoplasm of other connective and soft tissue of pelvis	1 (0.1)
		C49.8, Malignant neoplasm of other connective and soft tissue, overlapping lesion of connective and soft tissue	1 (0.1)
	C64-C68, Malignant neoplasms of urinary organs		1 (0.1)
	C69-C72, Malignant neoplasms of eye, brain, and other parts of central nervous system		13 (1.9)
		C71.5, Malignant neoplasm of ventricle	1 (0.1)
		C71.7, Malignant neoplasm of brain stem	3 (0.4)
		C71.8, Malignant neoplasm of overlapping lesion of brain	3 (0.4)
		C71.9, Malignant neoplasm of brain, unspecified	4 (0.6)
		C72.0, Malignant neoplasm of spinal cord	1 (0.1)
		C72.9, Malignant neoplasm of central nervous system, unspecified	1 (0.1)
	C73-C75, Malignant neoplasms of thyroid and other endocrine glands		2 (0.3)
		C74.9, Malignant neoplasm of adrenal gland, unspecified	1 (0.1)
		C75.3, Malignant neoplasm of pineal gland	1 (0.1)
	C81-C96, Malignant neoplasms, stated or presumed to be primary, of lymphoid, haematopoietic and related tissue		4 (0.6)
		C85.2, Mediastinal (thymic) large B-cell lymphoma	1 (0.1)
		C91.00, Acute lymphoblastic leukaemia [ALL]	1 (0.1)
		C92.00, Acute myeloblastic leukaemia [AML]	2 (0.3)
	D10-D36, Benign neoplasms		2 (0.3)
		D33, Benign neoplasm of brain and other parts of central nervous system	1 (0.1)
		D33.4, Benign neoplasm of spinal cord	1 (0.1)

III Diseases of the blood and blood-forming organs and certain disorders involving the immune mechanism	5 (0.7)
D55-D59, Hemolytic anemias	1 (0.1)
D57.0, Sickle-cell anemia with crisis	1 (0.1)
D60-D64, Aplastic and other anemias	1 (0.1)
D61.0, Constitutional aplastic anemia	1 (0.1)
D70-D77, Other diseases of blood and blood-forming organs	2 (0.3)
D76.1, Hemophagocytic lymphohistiocytosis	2 (0.3)
D80-D90, Certain disorders involving the immune mechanism	1 (0.1)
D82.1, DiGeorge syndrome	1 (0.1)
IV Endocrine, nutritional and metabolic diseases	77 (11.5)
E50-E64, Other nutritional deficiencies	2 (0.3)
E51.8, Other manifestations of thiamine deficiency	1 (0.1)
E61.5, Molybdenum deficiency	1 (0.1)
E70-E90, Metabolic disorders	75 (11.2)
E71.1, Other disorders of branched-chain amino-acid metabolism	1 (0.1)
E71.3, Disorders of fatty-acid metabolism	1 (0.1)
E72.0, Disorders of amino-acid transport	1 (0.1)
E72.4, Disorders of ornithine metabolism	1 (0.1)
E72.5, Disorders of glycine metabolism	4 (0.6)
E72.8, Other specified disorders of amino-acid metabolism	2 (0.3)
E75.0, GM2 gangliosidosis	1 (0.1)
E75.1, Other gangliosidosis	2 (0.3)
E75.2, Other sphingolipidosis	26 (3.9)
E75.4, Neuronal ceroid-lipofuscinosis	12 (1.8)
E76.0, Mucopolysaccharidosis type I	2 (0.3)
E76.2, Other mucopolysaccharidoses	10 (1.5)
E76.3, Mucopolysaccharidosis, unspecified	2 (0.3)
E77.0, Defects of lysosomal enzyme posttranslational modification	2 (0.3)
E79.8, Other disorders of purine and pyrimidine metabolism	2 (0.3)
E83.0, Disorders of copper metabolism	3 (0.4)
E84.87, Cystic fibrosis with other multiple manifestations	1 (0.1)
E88.8, Other specified metabolic disorders	1 (0.1)
V Mental and behavioural disorders	7 (1.0)
F80-F89, Disorders of psychological development	7 (1.0)
F84.2, Rett syndrome	7 (1.0)
VI Diseases of the nervous system	181 (27.1)
G00-G09, Inflammatory diseases of the central nervous system	7 (1.0)
G04.8, Other encephalitis, myelitis, and encephalomyelitis	1 (0.1)
G04.9, Encephalitis, myelitis, and encephalomyelitis, unspecified	1 (0.1)
G09, Sequelae of inflammatory diseases of central nervous system	5 (0.7)
G10-G14, Systemic atrophies primarily affecting the central nervous system	20 (3.0)
G10, Huntington's disease	2 (0.3)

G11.1, Early-onset cerebellar ataxia	2 (0.3)
G11.3, Cerebellar ataxia with defective DNA repair system	1 (0.1)
G11.8, Other hereditary ataxias	1 (0.1)
G12.0, Infantile spinal muscular atrophy, type I [Werdnig-Hoffmann type]	8 (1.2)
G12.1, Other inherited spinal muscular atrophy	5 (0.7)
G12.9, Spinal muscular atrophy, unspecified	1 (0.1)
G20-G26, Extrapyramidal and movement disorders	1 (0.1)
G23.0, Hallervorden-Spatz disease	1 (0.1)
G30-G32, Other degenerative diseases of the nervous system	26 (3.9)
G31.0, Focal brain atrophy	3 (0.4)
G31.81, Mitochondrial cytopathy	4 (0.6)
G31.88, Other specified degenerative diseases of nervous system	9 (1.3)
G31.9, Degenerative disease of nervous system, unspecified	9 (1.3)
G32.8, Other specified degenerative diseases of nervous system in diseases classified elsewhere	1 (0.1)
G40-G47, Episodic and paroxysmal disorders	31 (4.6)
G40.3, Generalized idiopathic epilepsy and epileptic syndromes	5 (0.7)
G40.4, Other generalized epilepsy and epileptic syndromes	16 (2.4)
G40.5, Special epileptic syndromes	7 (1.0)
G40.6, Grand mal seizures, unspecified (with or without petit mal)	1 (0.1)
G40.8, Other epilepsies	1 (0.1)
G47.32, Sleep-related hypoventilation syndrome	1 (0.1)
G50-G59, Nerve, nerve root, and plexus disorders	1 (0.1)
G57.8, Other mononeuropathies of lower limb	1 (0.1)
G70-G73, Diseases of myoneural junction and muscle	30 (4.5)
G70.9, Myoneural disorder, unspecified	2 (0.3)
G71.00, Muscular dystrophy (Duchenne)	15 (2.2)
G71.2, Congenital myopathies	10 (1.5)
G71.3, Mitochondrial myopathy, not elsewhere classified	1 (0.1)
G71.8, Other primary myopathies	1 (0.1)
G71.08, other muscular dystrophies	1 (0.1)
G80-G83, Cerebral palsy and other paralytic syndromes	1 (0.1)
G80.0, Spastic quadriplegic cerebral palsy	1 (0.1)
G90-G99, Other disorders of the nervous system	64 (9.6)
G92.9, Other and unspecified toxic encephalopathy	1 (0.1)
G93.1, Anoxic brain damage, not elsewhere classified	31 (4.6)
G93.4, Encephalopathy, unspecified	27 (4.0)
G93.9, Disorder of brain, unspecified	1 (0.1)
G94.3, Encephalopathy in other diseases classified elsewhere	3 (0.4)
G96.8, Other specified disorders of central nervous system	1 (0.1)
IX Diseases of the circulatory system	8 (1.2)
I30-I52, Other forms of heart disease	2 (0.3)
I42.0, Dilated cardiomyopathy	2 (0.3)
I60-I69, Cerebrovascular diseases	6 (0.9)

I69.1, Sequelae of intracerebral hemorrhage	3 (0.4)
I69.3, Sequelae of cerebral infarction	3 (0.4)
XI Diseases of the digestive system	7 (1.0)
K50-K52, Noninfective enteritis and colitis	2 (0.3)
K50.82, Crohn's disease of both small and large intestine, multiple sites	2 (0.3)
K80-K87, Disorders of gallbladder, biliary tract and pancreas	1 (0.1)
K85.91, Acute pancreatitis, unspecified: With organ failure	1 (0.1)
K90-K93, Other diseases of digestive system	4 (0.6)
K91.2, Postsurgical malabsorption, not elsewhere classified	4 (0.6)
XIII Diseases of the musculoskeletal system and connective tissue	3 (0.4)
M30-M36, Systemic connective tissue disorders	2 (0.3)
M30.1, Polyarteritis with lung involvement [Churg-Strauss]	1 (0.1)
M32.1, Systemic lupus erythematosus with organ or system involvement	1 (0.1)
M86-M90, Other osteopathies	1 (0.1)
M86.97, Osteomyelitis, unspecified: Ankle and foot [tarsus, metatarsus, phalanges, ankle, other joints of foot]	1 (0.1)
XVI Certain conditions originating in the perinatal period	124 (18.5)
P00-P04, Fetus and newborn affected by maternal factors and by complications of pregnancy, labour and delivery	1 (0.1)
P01.5, Fetus and newborn affected by multiple pregnancy	1 (0.1)
P10-P15, Birth trauma	3 (0.4)
P10.9, Unspecified intracranial laceration and haemorrhage due to birth injury	3 (0.4)
P20-P29, Respiratory and cardiovascular disorders specific to the perinatal period	1 (0.1)
P27.1, Bronchopulmonary dysplasia originating in the perinatal period	1 (0.1)
P50-P61, Hemorrhagic and hematological disorders of fetus and newborn	3 (0.4)
P57.9, Kernicterus, unspecified	1 (0.1)
P52.9, Intracranial (nontraumatic) hemorrhage in fetus and newborn, unspecified	2 (0.3)
P90-P96, Other disorders originating in the perinatal period	116 (17.3)
P91.2, Cerebral leukomalacia in newborn	5 (0.7)
P91.6, Hypoxic-ischemic encephalopathy in newborn [HIE]	76 (11.4)
P91.8, Other specified cerebral disorders in newborn	3 (0.4)
P91.9, Cerebral disorder in newborn, unspecified	1 (0.1)
P96.8, Other specified conditions originating in the perinatal period	31 (4.6)
XVII Congenital malformations, deformations and chromosomal abnormalities	212 (31.7)
Q00-Q07, Congenital malformations of the nervous system	64 (9.6)
Q03.8, Other congenital hydrocephalus	1 (0.1)
Q03.9, Congenital hydrocephalus, unspecified	1 (0.1)
Q04.2, Holoprosencephaly syndrome	3 (0.4)
Q04.3, Other reduction deformities of brain	27 (4.0)
Q04.4, Septo-optic dysplasia	1 (0.1)
Q04.6, Congenital cerebral cysts	1 (0.1)
Q04.8, Other specified congenital malformations of brain	14 (2.1)

Q04.9, Congenital malformation of brain, unspecified	3 (0.4)
Q05.2, Lumbar spina bifida with hydrocephalus	2 (0.3)
Q05.3, Sacral spina bifida with hydrocephalus	1 (0.1)
Q05.4, Spina bifida, unspecified, with hydrocephalus	1 (0.1)
Q05.7, Lumbar spina bifida without hydrocephalus	1 (0.1)
Q05.9, Spina bifida, unspecified	6 (0.9)
Q07.0, Arnold-Chiari syndrome	1 (0.1)
Q02, Microcephaly	1 (0.1)
Q20-Q28, Congenital malformations of the circulatory system	6 (0.9)
Q20.0, Common arterial trunk	1 (0.1)
Q23.4, Hypoplastic left heart syndrome	1 (0.1)
Q24.8, Other specified congenital malformations of heart	1 (0.1)
Q25.7, Other congenital malformations of pulmonary artery	1 (0.1)
Q28.28, Other congenital arteriovenous malformations of cerebral vessels	1 (0.1)
Q28.3, Other malformations of cerebral vessels	1 (0.1)
Q30-Q34, Congenital malformations of the respiratory system	2 (0.3)
Q32.0, Congenital tracheomalacia	2 (0.3)
Q38-Q45, Other congenital malformations of the digestive system	1 (0.1)
Q44.2, Atresia of bile ducts	1 (0.1)
Q60-Q64, Congenital malformations of the urinary system	1 (0.1)
Q60.4, Bilateral renal hypoplasia	1 (0.1)
Q65-Q79, Congenital malformations and deformations of the musculoskeletal system	10 (1.5)
Q68.8, Other specified congenital musculoskeletal deformities	1 (0.1)
Q74.3, Arthrogryposis multiplex congenita	2 (0.3)
Q74.8, Other specified congenital malformations of limbs	1 (0.1)
Q77.2, Short rib syndrome	2 (0.3)
Q78.2, Osteopetrosis	2 (0.3)
Q79.3, Gastroschisis	1 (0.1)
Q79.6, Ehlers-Danlos syndrome	1 (0.1)
Q80-Q89, Other congenital malformations	62 (9.3)
Q81.0, Epidermolysis bullosa simplex	4 (0.6)
Q81.1, Epidermolysis bullosa atrophicans gravis	1 (0.1)
Q81.2, Epidermolysis bullosa dystrophica	11 (1.6)
Q81.8, Other epidermolysis bullosa	2 (0.3)
Q81.9, Epidermolysis bullosa, unspecified	2 (0.3)
Q82.9, Congenital malformation of skin, unspecified	1 (0.1)
Q85.1, Tuberous sclerosis (of brain)	1 (0.1)
Q86.0, Alcohol embryopathy (with dysmorphic features)	1 (0.1)
Q87.0, Congenital malformation syndromes predominantly associated with facial anomalies	7 (1.0)
Q87.1, Congenital malformation syndromes predominantly affecting growth	7 (1.0)
Q87.2, Congenital malformation syndromes with predominant involvement of limbs	1 (0.1)
Q87.5, Other congenital malformation syndromes affecting predominantly musculoskeletal system	1 (0.1)

Q87.8, Other specified congenital malformation syndromes, not elsewhere classified	21 (3.1)
Q89.9, Congenital malformation, unspecified	2 (0.3)
Q90-Q99, Chromosomal abnormalities, not elsewhere classified	66 (9.9)
Q90.9, Down syndrome, unspecified	10 (1.5)
Q91.0, Trisomy 18, meiotic nondisjunction	1 (0.1)
Q91.2, Trisomy 18, translocation	1 (0.1)
Q91.3, Edwards syndrome, unspecified	5 (0.7)
Q91.4, Trisomy 13, meiotic nondisjunction	1 (0.1)
Q91.7, Patau syndrome, unspecified	2 (0.3)
Q92.1, Complete trisomy, mosaic (mitotic nondisjunction)	2 (0.3)
Q92.2, Partial trisomy, major type	2 (0.3)
Q92.3, Partial trisomy, minor type	1 (0.1)
Q92.8, Other specified trisomies and partial trisomies of autosomes	3 (0.4)
Q93.0, Complete monosomy, meiotic nondisjunction	1 (0.1)
Q93.3, Deletion of short arm of chromosome 4	1 (0.1)
Q93.5, Other deletions of part of a chromosome	11 (1.6)
Q93.8, Other deletions of autosomes	2 (0.3)
Q96.9, Turner syndrome, unspecified	1 (0.1)
Q99.8, Other specified chromosome abnormalities	21 (3.1)
Q99.9, Chromosome abnormality, unspecified	1 (0.1)
XIX Injury, poisoning and certain other consequences of external causes	6 (0.9)
S00-S09, Injuries to the head	4 (0.6)
S00.85, Superficial injury of other parts of head: Contusion	1 (0.1)
S06.1, Traumatic cerebral oedema	1 (0.1)
S06.20, Diffuse brain and cerebellar injury, unspecified	1 (0.1)
S06.9, Intracranial injury, unspecified	1 (0.1)
T80-T88, Complications of surgical and medical care, not elsewhere classified	1 (0.1)
T86.02, Acute graft-versus-host disease, grade III and IV	1 (0.1)
T90-T98, Sequelae of injuries, of poisoning, and of other consequences of external causes	1 (0.1)
T94.0, Sequelae of injuries of multiple body regions	1 (0.1)
